# Effect of *Zingiber officinale* R. rhizomes (ginger) on pain relief in primary dysmenorrhea: a placebo randomized trial

**DOI:** 10.1186/1472-6882-12-92

**Published:** 2012-07-10

**Authors:** Parvin Rahnama, Ali Montazeri, Hassan Fallah Huseini, Saeed Kianbakht, Mohsen Naseri

**Affiliations:** 1 Department of Midwifery, Herbal Research Center, Shahed University, Tehran, Iran; 2 Mental Health Research Group, Health Metrics Research Center, Iranian Institute for Health Sciences Research, ACECR, Tehran, Iran; 3 Department of Pharmacology, Research Institute of Medicinal Plants, ACECR, Karaj, IR, Iran; 4 Traditional Iranian Medicine Clinical Trial Research Center, Shahed University, Tehran, Iran

## Abstract

**Background:**

Z*ingiber officinale* R. rhizome (ginger) is a popular spice that has traditionally been used to combat the effects of various inflammatory diseases. The aim of this study was to evaluate the effects of ginger on pain relief in primary dysmenorrhea.

**Method:**

This was a randomized, controlled trial. The study was based on a sample of one hundred and twenty students with moderate or severe primary dysmenorrhea. The students were all residents of the dormitories of Shahed University. They were randomly assigned into two equal groups, one for ginger and the other for placebo in two different treatment protocols with monthly intervals. The ginger and placebo groups in both protocols received 500 mg capsules of ginger root powder or placebo three times a day. In the first protocol ginger and placebo were given two days before the onset of the menstrual period and continued through the first three days of the menstrual period. In the second protocol ginger and placebo were given only for the first three days of the menstrual period. Severity of pain was determined by a verbal multidimensional scoring system and a visual analogue scale.

**Results:**

There was no difference in the baseline characteristics of the two groups (placebo n = 46, ginger n = 56). The results of this study showed that there were significant differences in the severity of pain between ginger and placebo groups for protocol one (P = 0.015) and protocol two (P = 0.029). There was also significant difference in duration of pain between the two groups for protocol one (P = 0.017) but not for protocol two (P = 0.210).

**Conclusion:**

Treatment of primary dysmenorrhea in students with ginger for 5 days had a statistically significant effect on relieving intensity and duration of pain.

**Trial registration:**

IRCT201105266206N3

## Background

Primary dysmenorrhea is the term for painful menstrual cramping, there is no pathological evidence for the condition and it occurs in up to 50% of menstruating females [1]. It can lead to a woman’s failure to function normally during menstruation rendering them unable to perform daily activities; it can contribute to absenteeism from work or school [2].

One reason that has been suggested as an explanation for primary dysmenorrhea is an increased production of uterine prostaglandins derived from cyclooxygenase (COX)-2 activities [3,4]. Studies have shown that an inhibition of prostaglandin synthesis occurs through inhibition of COX-2 that could be exerted by nonspecific nonsteroidal anti-inflammatory drugs (NSAIDs). These drugs have useful effects such as anti-inflammatory, antipyretic and analgesic [5,6]. Moreover studies have indicated that the conventional treatment for primary dysmenorrhea has a failure rate of 20% to 25% [7]. These procedures may be contradictory or not tolerated by some women with primary dysmenorrhea [8]. Given the contraindications and side effects of NSAIDs as well as their limited efficacy, an investigation of alternative treatments with low toxicity such as herbal products is warranted.

Ginger has a long history of traditional use. It contains several constituents such as gingerol, gingerdiol, and gingerdione, beta-carotene, capsaicin, caffeic acid and curcumin [9,10].

Several studies have demonstrated that ginger has beneficial effects to cancer prevention [11], pregnancy- related nausea and vomiting [12], chemotherapy nausea [13], nausea and vomiting after surgery [14] and osteoarthritis [15]. It has been shown that ginger acts as an inhibitor on cyclooxygenase (COX) and lipooxygenase [16], resulting in an inhibition of leukotriene [17] and prostaglandin [18] synthesis. Therefore ginger has been used as an anti-inflammatory acting by inhibition of prostaglandin synthesis [19]. Ginger is therefore worthy of consideration as an analgesic in primary dysmenorrhea. Other research on the effects of ginger on dysmenorrhea has been of limited value as the study was not randomized and there was no placebo group [18]. The present clinical trial was undertaken to investigate the effect of 1500 mg ginger daily on pain relief in students with moderate to severe primary dysmenorrha, when treated before and at the onset of the menstrual period or treated only at the onset of the menstrual period.

## Methods

### Trial design

The study was a double blind, placebo-controlled and parallel-group study with balanced randomization [1:1] for the two groups. The study was conducted in Tehran, Iran from June 2008 to December 2008.

### Participants

A sample of female students aged 18 and over was selected for the trial. Inclusion criteria consisted of: being single, having a menstrual cycle that lasts from 21 to 35 days with 2 to 6 days of flow and average blood loss of 20 to 60 ml [20], with moderate to severe primary dysmenorrha. The severity of dysmenorrhea was determined by a verbal multidimensional scoring system [21], with four grades as follows: painless menstruation = 0, menstruation with pain but rare use of analgesics or limit to normal activity = 1, menstruation with moderate pain with influence on daily activity and use of analgesics for pain relief = 2, and menstruation with severe pain with significant limitation to daily activity, ineffective use of analgesics, and such symptoms as headache, tenderness, nausea, vomiting , and diarrhea = 3. Patients with moderate to severe dysmenorrhea (scores of 2 or 3) were included. The criteria that determined exclusion from the study were as follows: diagnoses of a disease, a history of pregnancy or taking oral contraceptives, body mass index (BMI) less than 19 kg/m^2^ or greater than 25 kg/m^2^, and mild dysmenorrhea.

### Study setting

The study was conducted at Shahed University dormitories from June 2008 to December 2008 in Tehran, Iran.

### Intervention

*Zingiber officinale* R. rhizomes were collected in April 2008 from the Iranian Institute of Medicinal Plants (affiliated to Iranian Academic Center for Education, Culture and Research-ACECR) field. A voucher specimen of the plant (number 1483) has been deposited in the Central Herbarium of the institute. The rhizomes were dried in a dark condition at room temperature and ground to a powder that was encapsulated.

### Preparation of Ginger and placebo capsules

The placebo capsules contained toast powder. The capsules were similar in shape, taste and color but one set contained 500 mg ginger powder per capsule and the others were placebo capsules. The ginger capsules did not have distinguishable smell. The capsules were prepared in the Institute of Medicinal Plants and put into coded packages. Capsules and their packages were identical in appearance.

After confirmation of a patient’s eligibility and after written informed consent was given, the students were randomly divided into two equal groups to be administered with ginger or placebo. The ginger and placebo groups received 500 mg capsules of ginger powder and placebo respectively three times a day in two different treatment protocols. Both treatment protocols were given at monthly intervals as follows:

*Treatment protocol 1:* In this protocol ginger and placebo were given two days before the onset of the menstrual period and continued through the first three days of the menstrual period.

**Figure 1:**
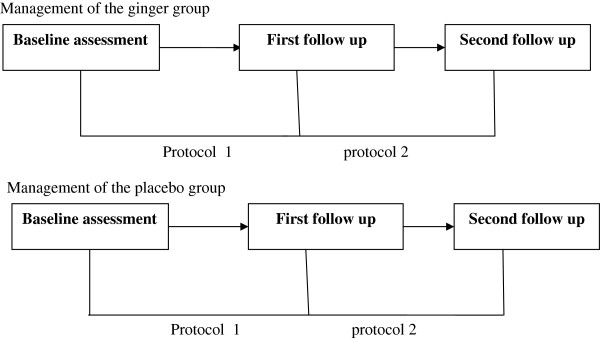
A schematic view of sequences of ginger and placebo protocols.

*Treatment protocol 2:* In this protocol ginger and placebo were given only for the first three days of the menstrual period (Figure 1).

In this study the two groups were independent and remained on the same allocation throughout the study.

### Outcomes

Severity and duration of pain were outcome measures for the study. The severity of pain was assessed before and after intervention by a visual analogue scale [22]. The visual analog scale (VAS) is a tool widely used to measure pain. Students were asked to indicate a perception of pain intensity (most commonly) along a 10 cm horizontal line. Duration of pain was determined by asking each student to indicate the number of hours she had experienced pain during the first three days of the menstrual period.

### Assessment of adverse effects

Students in both groups were requested to write down and report any unwanted adverse effects including diarrhea, upset stomach and heartburn. In addition, changes in menstrual cycles including duration of menses and interval of cycles were asked.

### Sample size

In order to demonstrate a significant difference between ginger and placebo groups using the pain severity test, the estimated sample size was calculated to be at least 50 students per group. A study with such a sample size would have a power of 90% at a 0.05 significance level.

### Randomization

**Figure 2:**
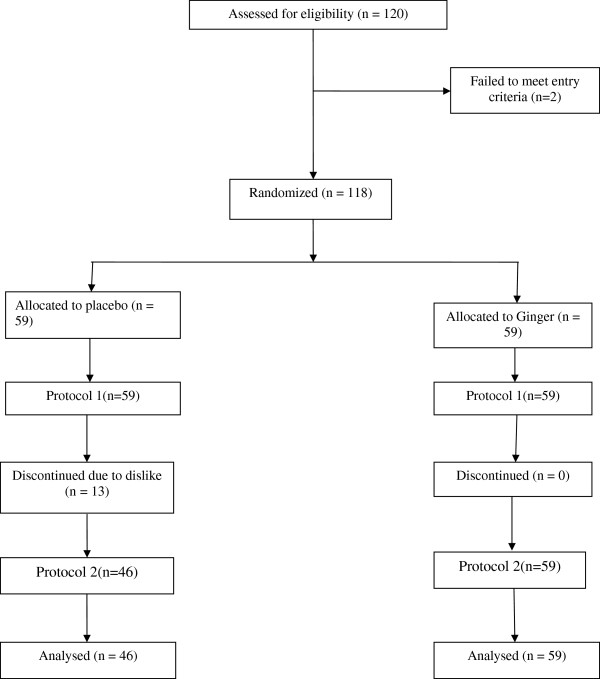
The trial flowchart.

A random numbers table was used for assigning participants in a 1:1 ratio to receive placebo and ginger using a block of two. An odd number was assigned to one patient and an even number to the other patient in each block. For each individual student recruited in the trial, a coded package was used (Figure 2).

### Allocation concealment

The randomization code was available only to the midwife who had not participated in the process of patient recruitment. The code was disclosed to the researchers when the statistical analysis had been completed by researchers.

### Blinding

This was a double- blind trial. Both the students and midwife providing care were blind to the treatment allocation. For this purpose, coded packages containing ginger and placebo capsules were used. The ginger and placebo capsules were identical in appearance, color and taste.

### Analysis

The SPSS version 16.0 was used to analyze the data. Descriptive analyses were carried out to explore the data. Analysis of covariance was performed for comparison of means for severity of pain and duration of pain in the two groups with baseline scores as the covariates. The Chi- square test was used to compare categorical variables, t test to compare pain score (OR severity of pain) and duration of pain between two groups, P value ≤ 0.05 was considered as significant.

### Ethics

The ethics committee of Shahed University approved the protocol. We obtained written informed consent from participants after comprehensive explanation of procedure involved.

## Results

The study involved a total of 105 students. Thirteen students who had received placebo discontinued the trial before completing the evaluation due to the fact that they indicated did not like to be involved in this research project any longer. More information on reasons for leaving was not captured. However, there were no significant differences between characteristics of 13 patients who left the placebo group and those who remained in the study (Table 1).

**Table 1:** Baseline demographics of the patients with primary dysmenorrhea in ginger and placebo groups

	**Ginger (n = 59)**	**Placebo (n = 46)**	Placebo* (n = 13)		
	**Mean (SD)**	**Mean (SD)**	**Mean (SD)**	**P**^**a**^	**P**^**b**^
Age (years)	21.4 (2.0)	21.3 (2.2)	21.2 (2.5)	0.244	0.883
Body Mass Index	20.4 (2.2)	20.7 (2.2)	20.3 (2.3)	0.371	0.603
Menarche (age in years)	13.7 (0.9)	13.6 (1.1)	14.0 (1.1)	0.638	0.241
Interval of cycles (days)	28.9 (2.2)	27.7 (2.7)	28.3 (1.8)	0.096	0.396
Duration of menses (hours)	6.6 (1.1)	6.7 (1.7)	6.7 (1.4)	0.640	0.949
Severity of pain (cm)	7.3 (1.1)	7.5 (0.9)	7.4 (1.58)	0.347	0.275
Duration of pain	19.38 (16.35)	19.06 (22.73)	18.15 (26.87)	0.170	0.912
Pain severity based on verbal multidimensional scoring system (number, %)				0.300	0.735
Mild	0 (0)	0 (0)	0 (0)		
Moderate	21 (35.6)	12 (26.1)	4 (30.8)		
Severe	38 (64.4)	34 (73.9)	9 (89.2)		

* Discontinued the trial.

^a^ Comparing ginger and placebo groups.

^b^ Comparing placebo and discontinued placebo group.

There were no significant differences between the two groups concerning baseline characteristics including age, BMI (Body Mass Index) and menstruation characteristics (Table 1).

The results of this study showed that there were significant differences in severity of pain between ginger and placebo groups for protocol one (P = 0.015, Table 2) and protocol two (P = 0.029, Table 3). When the analysis was adjusted for baseline score the results still remained significant for both protocols (protocol 1: P = 0.003, Table 2; protocol 2: P = 0.008, Table 3).

**Table 2:** Comparison of pain severity and duration of pain between two groups in protocol 1

	**Baseline**		**Protocol 1**		**Mean change in protocol 1**			
	**Ginger (n = 59)**	**Placebo (n = 46)**	**Ginger (n = 59)**	**Placebo (n = 46)**	**Ginger (n = 59)**	**Placebo (n = 46)**		
	**Mean (SD)**	**Mean (SD)**	**Mean (SD)**	**Mean (SD)**	**Mean (SD)**	**Mean (SD)**	**P**^**a**^	**P**^**b**^
Severity of pain	7.34 (1.1)	7.52 (0.93)	5.12 (2.69)	6.58 (2.02)	2.21 (2.87)	0.93 (2.24)	0.015	0.003
Duration of pain	19.38 (16.35)	19.06 (22.73)	14.7 (18.36)	21.36 (25.59)	4.59 (10.55)	-2.30 (18.24)	0.017	0.016

^a^ Derived from t-test.

^b^ Derived from ANCOVA adjusted for baseline.

**Table 3:** Comparison of pain severity and duration of pain between two groups in protocol 2

	**Baseline**		**Protocol 2**		**Mean change in protocol 2**			
	**Ginger (n = 59)**	**Placebo (n = 46)**	**Ginger (n = 59)**	**Placebo (n = 46)**	**Ginger (n = 59)**	**Placebo (n = 46)**		
	**Mean (SD)**	**Mean (SD)**	**Mean (SD)**	**Mean (SD)**	**Mean (SD)**	**Mean (SD)**	**P**^**a**^	**P**^**b**^
Severity of pain	7.34 (1.1)	7.52 (0.93)	4.61 (2.55)	6.01 (2.65)	2.72 (2.82)	1.51 (2.77)	0.029	0.008
Duration of pain	19.38 (16.35)	19.06 (22.73)	10.88 (14.54)	15.57 (14.72)	8.50 (17.77)	3.48 (23.08)	0.210	0.080

^a^ Derived from t-test.

^b^ Derived from ANCOVA adjusted for baseline.

There was also significant difference in duration of pain between the two groups for protocol one (P = 0.017, Table 2) but not for protocol two (P = 0.210, Table 3). The results also showed that there was significant difference between the two groups in duration of pain in protocol one (P = 0.016, Table 2) but not for protocol two (P = 0.080, Table 3) when the analysis was adjusted for baseline scores.

Overall the ginger group reported 11 h less pain duration than the placebo group. Similarly they reported 3 cm less in severity of pain than the placebo group.

In all, 3 students in the ginger group (5.1%) and 4 students in the placebo group (8.7%) reported adverse effects. Students in the ginger group reported heartburn while students in the placebo group reported nausea.

## Discussion

The results indicate that administration of 1500 mg ginger powder daily for three days is a safe and effective way to produce analgesia in students with primary dysmenorrhea.

Although the 1.4 and 2.0 point (cm) reduction in pain severity seen under protocol 1 and protocol 2 were statistically significant, as suggested if we consider a reduction of 3 point (cm) pain severity in the VAS as clinically significant [23], then one might argue that the finding was not clinically significant. However, the 3-point reduction was reported for other conditions and it is worth to establish this for ginger in the future studies.

Studies have shown that the NSAIDs are effective in treating dysmenorrhea comparing to placebo [24-26], but due to the absence of therapeutic response or intolerance to gastro- intestinal adverse events about 30% of women with primary dysmenorrhea would not take these drugs [27-29]. However, the result of this study showed that 5.1% students were experienced heartburn in ginger group and there were no significant differences between the two groups concerning to gastrointestinal adverse effects. Ginger is generally considered a safe herbal medicine [30].

The results of present study showed that taking ginger 2 days before the onset of the menstrual cycle was significantly better at decreasing the duration of pain. However, there was some indication that the magnitude of decrease in pain duration for protocol 2 was better than protocol one. The explanation for such an observation needs more investigation, although one might argue that this apparent superiority was due to under reporting by students using protocol 2.

Several factors including increased production of prostaglandins may be involved in menstrual pain [31]. To explain the effects of ginger on pain relief from dysmenorrhea, it has been reported that ginger inhibits cyclooxygenase and lipooxygenase pathways in prostaglandin and leukotriene synthesis [32] and the anti-inflammatory property of ginger has been attributed to inhibition of prostaglandin synthesis [19,33]. However the inhibition of prostaglandin synthesis may be the key mechanism for ginger’s effect on menstruation pain in this study.

The findings of this research are in accordance with a previous observation that compared efficacy of ginger to some NSAID drugs [18]. Similarly, another study was found that ginger had effect on pain relief in primary dysmenorrhea [34]. Finally, studies are needed in order to make ginger capsules in a standardized fashion and dose-finding studies are recommended.

## Limitations

This study is somewhat limited by a lack of observation regarding the effects of ginger on the other symptoms associated with dysmenorrhea. Furthermore, 13 patients from placebo group left the study. Unfortunately we did not collect data on reasons for leaving and this is a limitation. Moreover we did not analyze the gingerol or shogaol content of our ginger powder nor was it made in a standardized fashion. Consequently we do not know how much of key constituents were in the capsules making it hard to know what dose of other ginger capsule to give in the future and to determine if all the capsules were uniform in there gingerols/shogaol content. Lastly, it is worth noting that no measure of adherence or blinding was assessed. Consequently, effect sizes could be exaggerated if blinding was not achieved. Conversely if some of the participants did not take enough capsules or at the right times it could biases our results to the null. However, it was found that the effects of ginger are large enough to be clinically significant and to alleviate primary dysmenorrhea.

## Conclusion

The results suggest that ginger may be an effective and safe therapy for relieving pain in women with primary dysmenorrhea if administered at the onset or during the 3 days prior to menstruation. Furthermore studies with larger number of patients concerning the efficacy and safety of different doses of ginger as well as its effects on the other dysmenorrhea symptoms are suggested to supplement this research.

## Competing interests

The authors declare that they have no competing interests.

## Authors’ contributions

PR was the main investigator, designed the study, collected the data, and performed analysis. AM contributed to the analysis, critically evaluated the paper, and provided the final draft. HF prepared the materials for this study. SK helped the main investigator to writing the manuscript. MN helped the main investigator to design the study. All authors read and approved the final revision of the manuscript.

## Pre-publication history

The pre-publication history for this paper can be accessed here:

http://www.biomedcentral.com/1472-6882/12/92/prepub

## References

[B1] Dawood MY Primary dysmenorrhea: Advances in pathogenesis and management Obstet Gynecol 2006 108 428 441 10.1097/01.AOG.0000230214.26638.0c 16880317

[B2] Milsom I Minic M Dawood MY Akin MD Spann J Niland NF Squire R Comparison of the efficacy and safety of nonprescription doses of naproxen and naproxen sodium with ibuprofen, acetaminophen, and placebo in the treatment of primary dysmenorrhea: a pooled analysis of five studies Clin Ther 2002 24 1384 10.1016/S0149-2918(02)80043-1 12380631

[B3] Rosenwaks Z Seegar-Jones G Menstrual pain: its origin and pathogenesis J Reprod Med 1980 25 207 12 7001019

[B4] Bieglmayer C Hofer G Kainz C Reinthaller A Kopp B Janisch H Concentrations of various arachidonic acid metabolites in menstrual fluid are associated with menstrual pain and are influenced by hormonal contraceptives Gynecol Endocrinol 1995 9 307 312 10.3109/09513599509160464 8629459

[B5] Edwards JE Moore AR Mcquay HJ Rofecoxib for dysmenorroea: meta-analysis using individual patient data BMC Womens Health 2004 4 5 10.1186/1472-6874-4-5 15265230 PMC493273

[B6] Daniels SE Torri S Desjardins PJ Valdecoxib for Treatment of Primary Dysmenorrhea, A Randomized, Double-blind Comparison with Placebo and Naproxen J Gen Intern Med 2005 20 62 67 10.1111/j.1525-1497.2004.30052.x 15693930 PMC1490036

[B7] Zhu X Proctor M Bensoussan A Smith CA Wu E Chinese herbal medicine for primary dysmenorrhoea Cochrane Database Syst Rev 2007 17 CD005288 10.1002/14651858.CD005288.pub2 17943847

[B8] Proctor ML Murphy PA Herbal and dietary therapies for primary and secondary dysmenorrhoea Cochrane Database Syst Rev 2001 CD002124 10.1002/14651858.CD002124 11687013

[B9] Kikuzaki H Nakatani N Cyclic diarylheptanoids from rhizomes ofZingiber officinale Phytochemistry 1996 43 273 277 10.1016/0031-9422(96)00214-2

[B10] Schulick P Ginger, common spice and wonder drug 1996 3 Brattleboro (VT): Herbal Free Press Ltd

[B11] Lee SH Cekanova M Baek SJ Multiple mechanisms are involved in 6-gingerol-induced cell growth arrest and apoptosis in human colorectal cancer cells Mol Carcinog 2008 47 197 208 10.1002/mc.20374 18058799 PMC2430145

[B12] Pongrojpaw D Somprasit C Chanthasenanont A A randomized comparison of ginger and dimenhydrinate in the treatment of nausea and vomiting in pregnancy J Med Assoc Thai 2007 90 1703 1709 17957907

[B13] Ryan JL Heckler CE Roscoe JA Dakhil SR Kirshner J Flynn PJ Hickok JT Morrow GR Ginger (Zingiber officinale) reduces acute chemotherapy-induced nausea: a URCC CCOP study of 576 patients Support Care Cancer 2011 20 1479 1489 21818642 10.1007/s00520-011-1236-3 PMC3361530

[B14] Chaiyakunapruk N Kitikannakorn N Nathisuwan S Leeprakobboon K Leelasettagool C The efficacy of ginger for the prevention of postoperative nausea and vomiting: a meta-analysis Am J Obstet Gynecol 2006 194 95 99 10.1016/j.ajog.2005.06.046 16389016

[B15] Haghighi M Khalvat A Toliat T Jallaei S Comparing the effects of ginger (zingiber officinale) extract and ibuprofen on patients with osteoarthritis Arch Iran Med 2005 8 267 271

[B16] Mustafa T Srivastava KC Jensen KB Drug development: report 9. Pharmacology of ginger, Zingiber officinale J Drug Dev 1993 6 25 89

[B17] Kiuchi F Iwakami S Shibuya M Hanaoka F Sandawa U Inhibition of prostaglandin and leukotriene biosynthesis by gingeroles and diarylhepatanoids Chem Pharm Bull (Tokyo) 1992 40 187 191 10.1248/cpb.40.387 1606634

[B18] Ozgoli G Goli M Moattar F Comparison of Effects of Ginger, Mefenamic Acid, and Ibuprofen on Pain in Women with Primary Dysmenorrhea J Altern Complement Med 2009 15 129 132 10.1089/acm.2008.0311 19216660

[B19] Grzanna R Lindmark L Frondoza CG Ginger-An herbal medicinal product with broad anti-inflammatory actions J Med Food 2005 8 125 132 10.1089/jmf.2005.8.125 16117603

[B20] Paula JAH Benign diseases of female reproductive tract 2007 14 Philadelphia: Berek and Novak’s Gynecology, Lippincott William and Wilkins: Wolterrs Kluwer business 446

[B21] Andersch B Milsom I An epidemiologic study of young women with dysmenorrheal Am J Obstet Gynecol 1982 144 655 660 7137249 10.1016/0002-9378(82)90433-1

[B22] Carlsson AM Assessment of chronic pain. I. Aspects of the Reliability and Validity of the Visual Analogue Scale Pain 1983 16 87 101 10.1016/0304-3959(83)90088-X 6602967

[B23] Lee JS Hobden E Stiell IG Wells GA Clinically important change in the visual analog scale after adequate pain control Acad Emerg Med 2003 10 1128 1130 10.1111/j.1553-2712.2003.tb00586.x 14525749

[B24] Dawood MY Efficacy and safety of piroxicam-B-cyclodextrin (PBCD, Brexidol). Comparison studies with ibuprofen, naproxen sodium and placebo in the relief of moderate to severe abdominal pain associated with primary dysmenorrhea. The Brexidol Study Group Today’s Therapeutic Trends 1999 17 273 288

[B25] Zhang WY Li Wan Po A Efficacy of minor analgesics in primary dysmenorrhoea: a systematic review Br J Obstet Gynaecol 1998 105 780 9 10.1111/j.1471-0528.1998.tb10210.x 9692420

[B26] Morrison JC Ling FW Forman EK Bates GW Blake PG Vecchin TJ Linden CV O’Connell MJ Analgesic efficacy of ibuprofen for treatment of primary dysmenorrhea South Med J 1980 73 999 1002 10.1097/00007611-198008000-00014 6996107

[B27] Campbell MA McGrath PJ Use of medication by adolescents for the management of menstrual discomfort Arch Pediatr Adolesc Med 151 905 912 9308868 10.1001/archpedi.1997.02170460043007

[B28] Traversa G Walker A Ippolito F Caffari B Capurso L Dezi A Koch M Maggini M Alegiani S Raschetti R Gastroduodenal toxicity of different nonsteroidal antiinflammatory drugs Epidemiology 1995 6 49 54 10.1097/00001648-199501000-00010 7888445

[B29] Higuchi K Umegaki E Watanabe T Yoda Y Morita E Murano M Tokioka S Arakawa T Present status and strategy of NSAIDs-induced small bowel injury J Gastroenterol 2009 44 879 888 10.1007/s00535-009-0102-2 19568687

[B30] Weidner MS Sigwart K Investigation of the teratogenic potential of Zingiber officinale extract in the rat Reprod Tocicol 2000 15 75 80 10.1016/S0890-6238(00)00116-7 11137381

[B31] Speroff L Fritz MA Clinical gynecologic endocrinology and infertility 2005 7 Philadelphia: Lippincott Williams and Wilkins

[B32] Ali BH Blunden G Tanira MO Nemmar A Some phytochemical, pharmacological and toxicological properties of ginger (Zingiber officinaleRoscoe): a review of recent research Food Chem Toxicol 2008 46 409 420 10.1016/j.fct.2007.09.085 17950516

[B33] Kim JK Kim Y Na KM Surh YJ Kim TY [6]-Gingerol prevents UVB-induced ROS production and COX-2 expression in vitro and in vivo Free Radic Res 2007 41 603 614 10.1080/10715760701209896 17454143

[B34] Rahnama P Falah Hossini H Mohammadi K Modares M Khajavi Shojaei K Askari M Mozayani P The effects of Zingiber Officional R on primary dysmenorrhea J Med Plants 2010 9 81 86

